# A Robust PVDF-Assisted Composite Membrane for Tetracycline Degradation in Emulsion and Oil-Water Separation

**DOI:** 10.3390/nano11123201

**Published:** 2021-11-26

**Authors:** Huijun Li, Xin Xu, Jiwei Wang, Xuefeng Han, Zhouqing Xu

**Affiliations:** 1 College of Chemistry and Chemical Engineering, Henan Polytechnic University, Jiaozuo 454000, China; lihuijunxgy@hpu.edu.cn (H.L.); XuXinwx@home.hpu.edu.cn (X.X.); 211912020008@home.hpu.edu.cn (J.W.); 2 School of Safety Science and Engineering, Henan Polytechnic University, Jiaozuo 454000, China

**Keywords:** GO/NH_2_-MIL-101(Fe), PVDF membrane, glass fiber, tetracycline degradation, oil-water separation

## Abstract

Tetracycline (TC) contamination in water has progressively exacerbated the environmental crisis. It is urgent to develop a feasible method to solve this pollution in water. However, polluted water often contains oil. This paper reported a glass fiber (FG)-assisted polyvinylidene fluoride (PVDF) hybrid membrane with dual functions: high TC degradation efficiency in emulsion and oil-water separation. It can meet the catalytic degradation of tetracycline in complex water. This membrane was decorated by coating the glass fiber with PVDF solution containing hydrophilic graphene oxide hybridized NH_2_-MIL-101(Fe) particles. Moreover, due to its strong mechanical strength enhanced by the glass fiber, it can be reused as TC degradation catalysts for dozens of times without cracking. Thanks to the hydrophobicity of PVDF and the surface pore size of MOFs, the prepared membrane showed a good oil-water separation performance. Besides, the hydrophilic graphene oxide (GO) and NH_2_-MIL-101(Fe) improved the membrane’s anti-fouling performance, allowing it to be reused as the separation membrane. Therefore, the outstanding stability and recoverability of the membrane make it as a fantastic candidate material for large-scale removal of TC as well as oil-water separation application.

## 1. Introduction

As we all know, human life is inseparable from water, which is the source of life of all things. However, with the rapid development of industry and medical technology, various pollutants discharged by people make the water environment pollution more and more serious, and the pollution components are becoming more and more complex, which seriously threatens biodiversity. The focus of this study is to realize the degradation of tetracycline in sewage and oil-water separation.

As the blood of industry, the emission and leakage of oil will also pose a great threat to environmental sustainability. Therefore, the separation of oil-water mixture is always an urgent problem to be solved [1]. At present, there are many technologies for separating oil-water mixture, including gravity separation, dispersant, centrifugation, and flocculation. However, these methods have some disadvantages, such as high operating cost, complex equipment, high energy consumption, and secondary pollution, which severely limit their application in practice. Especially, these technologies are ineffective for treating emulsified oil in water with particle size less than 10 µM, thus the research of oil-water separation has become the focus of current researchers [2]. Among them, membrane separation technology is widely regarded as an effective method to separate oil-water mixture and emulsion, which has the advantages of low energy consumption and little environmental impact [3]. For example, Gao et al. [4] firmly adhered regenerated cellulose to PVDF membrane by supramolecular adhesive, and Alammar et al. [5] prepared graphene-based nanocomposite membrane to realize efficient oil-water separation.

In addition, tetracycline (TC), as the largest production and clinical use in the livestock and poultry breeding industry, has also been an increased risk of resistance in bacterial and antibiotic resistance in humans. TC can exist in water for a long time after being discharged into water. Therefore, the research on sewage treatment containing TC has become a hot spot of present researchers [6,7,8,9]. In this pursuit of stimulation, metal-organic frameworks, which are formed by the self-assembly of central metal ions and organic ligands, have attracted wide attention because of their unique properties [10,11,12]. In view of the superior regular and orderly pore structure and excellent catalysis properties, MOFs often showed good performance in removing antibiotics contaminants from water through the synergy of physical screening and chemical degradation ability [13,14]. Nevertheless, it is very difficult to collect MOFs powder from solution after reaction, which is the main problem. To address this practical problem, supporting powdery catalyst on a carrier can effectively convert the problems such as poor stability and difficult recovery. In conclusion, it is most satisfactory to select membrane material as a carrier, which can realize the comprehensive treatment of sewage with complex components [15,16].

As we know, TC is more soluble in organic phases [17,18,19]. At the same time, in order to better apply to the sewage containing organic phase in real life. The prepared membrane should have hydrophobicity thus having more opportunities to contact TC in the organic phase. Among the developed polymer membranes, polyvinylidene fluoride (PVDF) is one of the most attractive membrane materials due to the repeated unit of (-CH_2_CF_2_-), resulting in its strong hydrophobicity and high thermal stability as well as chemical resistance [20,21]. However, when a thing reaches its extreme, it reverses its course. The more hydrophobic the membrane is, the more likely it is to be contaminated. The adhered organic phases are hard to remove, decreasing the removal efficiency, therefore causing membrane waste [22,23,24]. Therefore, it is necessary to modify PVDF to obtain a membrane with certain hydrophobicity that is easy to recycle and resistant to organic contaminants. Another obstacle is that the membrane formed by pure PVDF as a carrier is too flexible, leading to its poor mechanical strength and easy to break in the degradation process. As a result, the objective of this study is to develop membranes with excellent anti-fouling properties, strong mechanical strength, good TC removal performance as well as easy recovery to meet the increasing environmental requirements.

Based on this notion, we reported herein a simple method to construct a glass fiber (FG)-assisted PVDF hybrid membrane with certain hydrophobicity and strong mechanical strength by coating the glass fiber with PVDF solution containing hydrophilic graphene oxide hybridized NH_2_-MIL-101(Fe) particles (named GO/NH_2_-MIL-101(Fe)/PVDF/FG membrane). As a derivative of graphene, GO is widely used in the treatment of environmental water samples because its surface contains a large number of hydroxyls, carboxyl, and epoxy groups, which is hydrophilic and have no pollution to the environment. For example, Ghanbarlou et al. [25] synthesized a novel nitrogen-doped graphene-iron-based electrocatalyst and Alammar et al. [26] engineered nanocomposite hydrogels based on sustainable cellulose acetate for water treatment. GO has abundant active sites and excellent dispersity, the introduction of which not only solve the problem of unstable adhesion of MOF particles, but also reduce the hydrophobicity of the membrane. On the other hand, introducing MOF into GO can improve the flux of GO membrane and the stability between GO and PVDF. Moreover, benefiting from the experience of building a house, incorporating steel bars into cement will strengthen the house. Similarly, adding chemically stable glass fiber in the process of membrane formation will availably increase the mechanical strength of the membrane. The application potential of the membrane was evaluated using pure water and emulsion. As anticipated, the prepared membrane showed good performance for the catalytic degradation of TC in the emulsion. Besides, its certain hydrophobicity can not only effectively avoid the problem of organic contamination, but also enough to achieve oil-water separation performance. Moreover, combined with the synergistic effect of porosity and surface pore size of MOFs, the prepared membrane displays better oil-water separation performance than the pure PVDF membrane. The synergistic use of the two functions of this membrane material can better remove tetracycline in oily sewage, and the characteristics of oil-water separation can also better purify water. This provides ideas for the treatment of water pollution caused by antibiotics and oil pollution in real life.

## 2. Materials and Methods

### 2.1. Materials

Polyvinylidene fluoride (PVDF), graphite powders, FeCl_3_·6H_2_O, *N*,*N*-Dimethylformamide (DMF), glass fiber (FG), *N*,*N*-Dimethylacetamide (DMA), p-Phthalic acid and 2-aminoterephthalic acid were purchased from Aladdin Chemistry Co., Ltd. (Shanghai, China). Anhydrous ethanol, sodium laurylsulfonate, trichloromethane, dichloromethane, p-benzoquinone, triethanolamine and toluene were purchased from Aladdin Industrial Corporation, China. All chemicals were analytical grade and can be used without further purification.

### 2.2. Preparation of Emulsion

Sodium laurylsulfonate (0.05 g) and gasoline (0.5 g), which were sufficiently dissolved in deionized water (1 L) and treated with ultrasonic for 2 h under room temperature to gain the oil-in-water emulsion [27]. As shown in Figure S1, there was no obvious change in 24 h or even 48 h, which proves that the emulsion structure was stable.

### 2.3. Preparation of Graphene Oxide

Preparation of graphene oxide (GO) by an improved method of Hummers [28,29]. In short, it was divided into 3 stages.

Low-temperature stage: dissolve 1.2 g graphite powder and 0.5 g sodium nitrate in 150 mL mixed acid solution (135 mL concentrated H_2_SO_4_ + 15 mL concentrated H_3_PO_4_), and stir for half an hour below 20 °C. Then, slowly add 7 g of potassium permanganate to keep the temperature of the solution below 20 °C and stir for 1 h.

Medium temperature stage: raise the temperature to 35 °C, stir for 2 h, and then slowly add the solution to 150 mL of ice water.

High-temperature stage: raise the temperature to 90 °C, after 20 min of continuous heating, the solution turns brown and produces red smoke. Stop heating and add 5 mL H_2_O_2_ (30%) dropwise with constant stirring until the solution turns golden yellow.

The resulting solution was allowed to stand overnight, the supernatant was decanted, the lower turbid liquid was centrifuged (12,000 rpm, 10 min), and the resulting solid was washed with 30% HCl until the supernatant, and the barium chloride solution were mixed without white precipitation. Next, wash the solids to neutrality with deionized water. The resulting solid was then vacuum dried at 50 °C for 12 h. The product was graphene oxide.

### 2.4. Preparation of GO/NH_2_-MIL-101(Fe)

The mesoporous GO/NH_2_-MIL-101(Fe) nanocomposite was prepared via a ‘one-pot’ method. After ultrasonic treatment for 20 min, 2-aminoterephthalic acid (0.494 g), and FeCl_3_·6H_2_O (1.62 g) were dissolved in DMF (72 mL). Then, transferred the solution to a 250 mL round-bottom flask, added GO (0.5 g), and heated in an oil bath at 110 °C for 20 h. After the reaction, it was cooled naturally to room temperature, and the solid powder was collected using a centrifuge after washing with ethanol and DMF for 3 times. Afterward, the collected brown powder was purified at 150 °C for 8 h.

### 2.5. Fabrication of Membranes

The above GO/NH_2_-MIL-101(Fe) (0.5 g) was ground, dispersed in DMA (20 mL), ultrasonically treated at 60 °C for 10 min, and the solid powder was uniformly dispersed. Then, in order to prevent agglomeration, PVDF particles (4 g) were added bit by bit to the above solution and stirred with a glass rod (1 h) until the solution became uniform and viscous, thus making a uniform coating solution. The prepared coating solution was evenly coated on one side of the glass fiber cloth (8 × 8 cm), immediately sprayed with deionized water to concentrate it into a membrane, and then dried in a 60 °C air-blast drying oven for 1 h. After taking it out, repeat the above operation on the other side, and finally, put it in a blast drying oven (60 °C) for 24 h to obtain a composite membrane.

### 2.6. Photocatalysis Experiment

A 2 cm × 2 cm membrane (the content of loaded MOFs was about 16 mg) and 2 drops of H_2_O_2_ (electron trapping agent for the classical photocatalytic experiment) were added to a 100 mg/L tetracycline aqueous solution or emulsion (100 mL). The photochemical reactor was kept in the dark and airtight environment for 1 h initially. Then, the reaction solution was located under a lamp, and samples were taken every 30 min. The whole reaction time was 3 h. The concentrations of tetracycline after the reaction were measured by an ultraviolet spectrophotometer.

### 2.7. Oil-Water Separation Experiment

Various organic phases, such as nitrobenzene, dichloromethane, chloroform, benzyl alcohol, toluene, cyclohexane, p-xylene, and gasoline, mixed with 40 mL water (stained with methylene blue) were used as models for oil-water separation experiment.

Oil flux calculation: F = V/(A × T);

Filtration efficiency calculation: μ = (V/V_0_) × 100%.

T represents the filtration time, V represents the volume of oil after filtration, A represents the surface area of the membrane, and V_0_ represents the volume of oil before filtration [30,31].

### 2.8. Characterizations of the Membrane

The morphology of the prepared membrane was characterized by Scanning Electron Microscopy (SEM, S-3400 N, Hitachi, Tokyo, Japan). The attenuated total reflection-Fourier transform infrared spectroscopy (ATR-FTIR) was conducted on a Nicolet iS50 system (Thermo Fisher Scientific, Waltham, MA, USA). The X-ray diffraction (XRD) data were collected by using an X-ray diffractometer (XRD, Bruker D8 Advance, Bruker AXS, Karlsruhe, Germany). X-ray photoelectron spectroscopy (XPS, Kratos AXIS Ultra DLD, SHIMADZU/KRATOS, Manchester, Britain) was performed to investigate the surface composition. The contact angles (CAs) of the membrane were tested by an OSA60 (LAUDA Scientific, Lauda-Königshofen, Germany).

## 3. Results

### 3.1. Results of Membrane Characterizations

**Figure 1:**
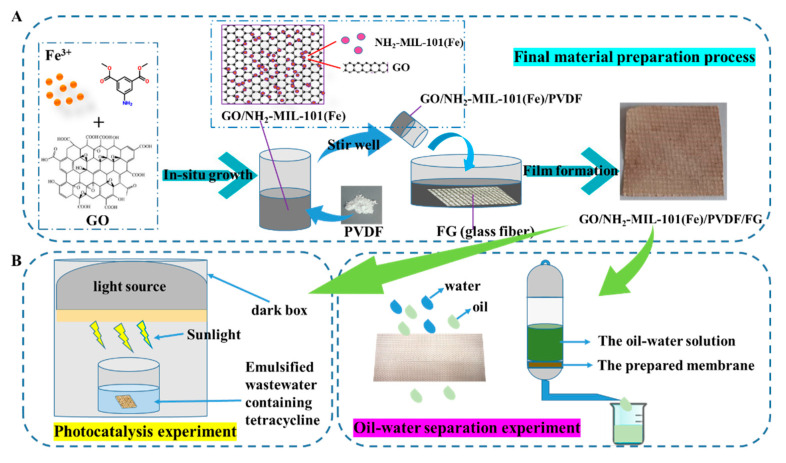
Schematic diagram of the preparation process of the final membrane (**A**) and the dual functions of tetracycline degradation and oil-water separation by on-site set-up illustration (**B**).

The photograph of the final membrane and the synthesis process is presented in Figure 1. The final membrane has a brown appearance caused by the existence of NH_2_-MIL-101(Fe) catalysts, and its surface is smooth and dense, both of which are beneficial for the following study.

**Figure 2:**
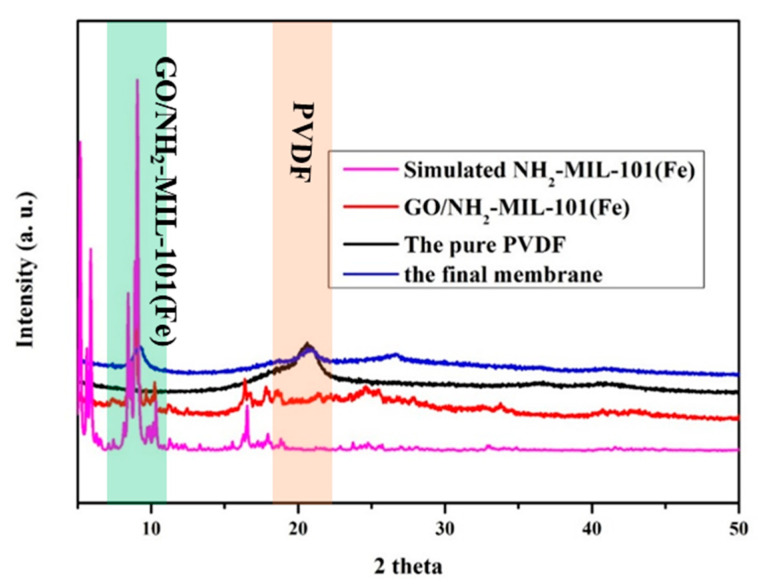
XRD patterns of the simulated NH_2_-MIL-101(Fe), GO/NH_2_-MIL-101(Fe), the pure PVDF, and the final membrane.

Figure 2 shows the XRD patterns of GO/NH_2_-MIL-101(Fe), PVDF, and the final membranes. The XRD pattern diffraction peaks of the original PVDF membrane appear at 20.11, which is consistent with the final membrane. Besides, GO/NH_2_-MIL-101(Fe)/PVDF/FG membrane still has the characteristic peak of NH_2_-MIL-101(Fe), which confirms that NH_2_-MIL-101(Fe) maintains a good crystal structure on the final membrane. Meanwhile, in the XRD pattern, the diffraction peaks of GO/NH_2_-MIL-101(Fe) are consistent with the typical diffraction peaks previously reported [32].

**Figure 3:**
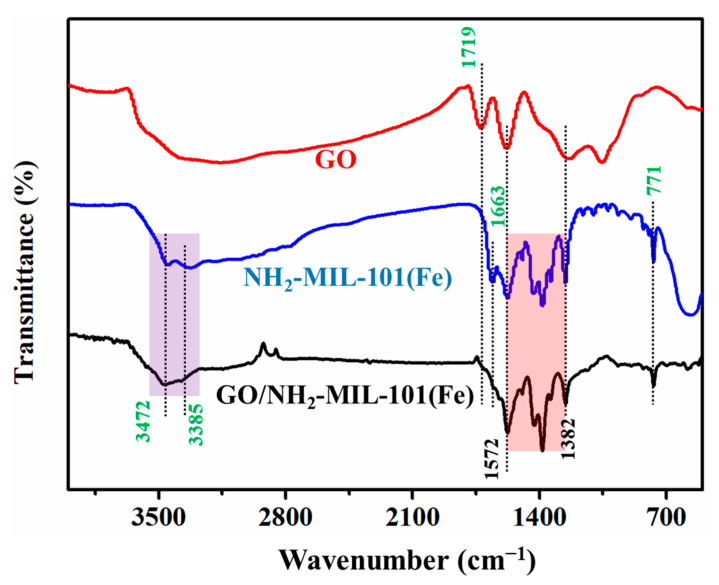
The IR spectra of GO, NH_2_-MIL-101 (Fe) and GO/NH_2_-MIL-101 (Fe).

In order to verify the interaction between NH_2_-MIL-101(Fe) and GO, their FTIR spectra were studied (Figure 3). It can be clearly seen that the main characteristic peaks of NH_2_-MIL-101(Fe) still exist in the infrared peaks of GO/NH_2_-MIL-101(Fe), such as the characteristic peak at 771 cm^−1^, and the characteristic peaks between 1572 cm^−1^ and 1382 cm^−1^ were highly consistent, which proves that the crystal structure has not changed during the synthesis process [33,34]. Besides, the doublet at 3472 cm^−^^1^ and 3385 cm^−1^ correspond with the –NH_2_ group, which proves that NH_2_-MIL-101(Fe) is successfully composited. Moreover, there is no characteristic peak of GO at 1719 cm^−1^ and no characteristic peak of NH_2_-MIL-101(Fe) at 1663 cm^−1^ in GO/NH_2_-MIL-101(Fe). Because NH_2_-MIL-101(Fe) may react with the –OH and –COOH groups on the surface of GO [35]. This shows that NH_2_-MIL-101(Fe) successfully grows on the appearance of GO. 

**Figure 4:**
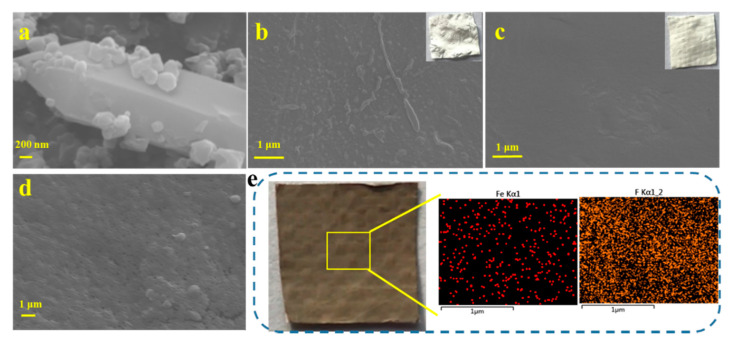
SEM images of the surface of (**a**) GO/NH_2_-MIL-101(Fe), (**b**) the pure PVDF membrane, (**c**) the membrane without GO/NH_2_-MIL-101(Fe), and (**d**) the final membrane; (**e**) the mapping results of the final membrane. The acceleration voltage was 10 kV. Gold spraying treatment was needed before observation.

As shown in Figure S2a, the original glass fiber has a porous surface and square pores in the membrane. When the PVDF layer was fixed on the membrane surface by the coagulation bath, almost no pores can be found on the surface. However, by comparing the PVDF/FG membrane with the pure PVDF membrane, it was found that the surface of PVDF/FG membrane was much denser (Figure S2b,c). The composition of GO/NH_2_-MIL-101(Fe)/PVDF/FG was studied by SEM. As shown in Figure 4, because of the existence of GO, the prepared GO/NH_2_-MIL-101(Fe) was evenly distributed on the membrane surface. Because of its strong adhesion and unique chemical properties, GO can be conveniently used as a connector for MOF materials anchoring [36]. Besides, the SEM image of the PVDF membranes without fibrous was also studied, which showed that the fiber-free composite membranes have an uneven surface and a low tensile strength and were unsuitable as repeated photocatalytic degradation and adsorption membranes (Figure 4b).

In order to evaluate the dispersion of GO/NH_2_-MIL-101(Fe) in the membrane matrix, elemental mapping was conducted. Figure 4e shows that Fe and F elements were uniformly distributed on the surface of elemental mapping images of the GO/NH_2_-MIL-101(Fe)/PVDF/FG membrane. According to the experimental results, GO/NH_2_-MIL-101(Fe) has good compatibility with PVDF, which may be caused by the interaction between the fluorine groups of PVDF and the plentiful functional groups onto GO/NH_2_-MIL-101(Fe). The interaction between CF_2_ segments in PVDF polymer and the carbonyl groups (–C=O) in GO will lead to the transformation of PVDF crystal phase [37,38].

In order to better prove our view, SEM micrographs of cross-sections of original PVDF membrane (a), glass fiber membrane (b), GO/PVDF/FG membrane (c), and GO/NH_2_-MIL-101(Fe)/PVDF membrane (d) are provided with three different resolutions, as depicted in Figure S3. According to Figure S3a, PVDF membrane has a sponge-like porous structure in cross-section, and its microstructure is tensile, thus the surface will wrinkle. Figure S3b shows the cross-section of glass fiber, and its morphology is similar to that of steel bars used in buildings, thus the membrane material prepared by using it as the skeleton has strong mechanical strength. Compared with FG membrane, there are many dopants on the glass fiber in the cross-section of GO/PVDF/FG membrane, which is similar to cement poured in buildings and firmly attached to the glass fiber. However, by comparing Figure S3c with Figure S3d, it is found that the GO layer on the surface of PVDF membrane reinforced with glass fiber is mostly flaky when it is modified with simple GO. Because of the hydrophilicity of GO, the flake GO attached to the membrane will easily fall off during contact with the water environment. However, this problem does not exist in GO/NH_2_-MIL-101(Fe)/PVDF membrane, and its dopant shows better adhesion with glass fiber.

**Figure 5:**
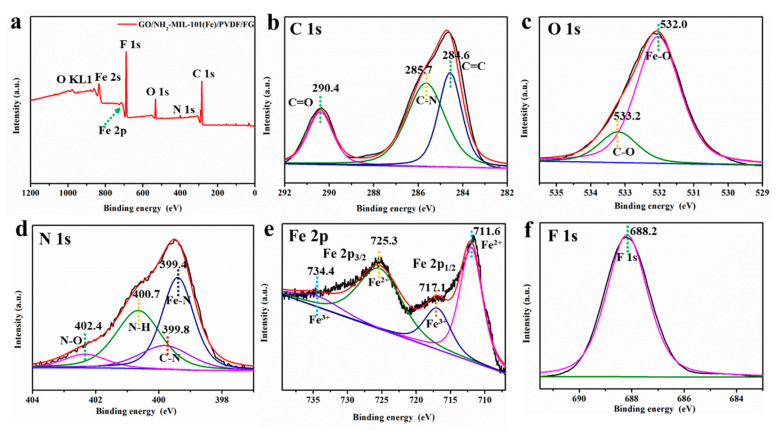
The XPS spectra (**a**) of GO/NH_2_-MIL-101(Fe)/PVDF/FG membrane, and (**b**) C 1s, (**c**) O 1s, (**d**) N 1s, (**e**) Fe 2p, and (**f**) F 1s of GO/NH_2_-MIL-101(Fe)/PVDF/FG membrane.

XPS was used to further analyzed the chemical environment of the GO/NH_2_-MIL-101(Fe) and GO/NH_2_-MIL-101(Fe)/PVDF/FG membranes. As shown in Figure S4, the binding energies of C=O, C-N and C=C in GO/NH_2_-MIL-101(Fe) show slight shifts by 0.3 eV, 0.2 eV, and 0.5 eV, respectively, to the high energy because of the interaction of GO and NH_2_-MIL-101(Fe) [39]. In addition, the binding energies of Fe 2p_3/2_ and Fe 2p_1/2_ display a negative shift suggesting the coordination of oxygen functional groups of GO with the unsaturated Fe sites resulting in a change of Fe oxidation states. Therefore, the above evidence demonstrates the composite material GO/NH_2_-MIL-101(Fe) is a complete whole and is not just physically mixed together [40]. The measurement scanning spectra of the GO/NH_2_-MIL-101(Fe)/PVDF/FG membrane is shown in Figure 5. It should be pointed out that the binding energies of C=O shift to a higher binding energy verifying the interaction between the composite PVDF and GO/NH_2_-MIL-101(Fe) [41]. Due to the interaction between GO and PVDF, the binding energy of Fe shifts to low energy, thus giving an increase in the oxidation state of the Fe centers in GO/NH_2_-MIL-101(Fe)/PVDF/FG membrane [42]. Combined with the above analysis results, we deem that we have successfully prepared the GO/NH_2_-MIL-101(Fe)/PVDF/FG membrane. 

### 3.2. Membrane Performance

#### 3.2.1. Mechanical Property

**Figure 6:**
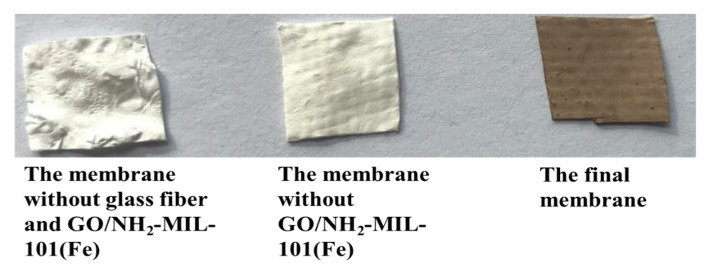
The photographs of different membranes.

For testing the effect of glass fiber and PVDF on mechanical properties of materials, the mechanical properties of the membranes with a length of 3 cm and width of 0.5 cm were inspected by an electronic single fiber strength tester [43,44]. As shown in Figure 6 and Table 1, five times the tension tests were carried out for PVDF membrane without glass fiber, PVDF membrane with glass fiber, and GO/NH_2_-MIL-101(Fe)/PVDF/FG membranes. The experimental results indicate that the average breaking strength of PVDF with glass fiber membrane was 0.0491 MPa, which was greatly larger than that of pure PVDF membrane without glass fiber (0.0245 MPa) (Video S1). Besides, after chelation with GO/NH_2_-MIL-101(Fe), the mechanical properties of the final membrane show a slightly rising trend with the breaking strength of 0.0495 MPa (Video S2). Moreover, when the final membrane was used as oil-water separation and photocatalytic materials several times, the breaking strengths almost did not change. The above results show that the final membrane loaded with NH_2_-MIL-101(Fe) catalysts equipped enough strength in the following applications.

#### 3.2.2. Photocatalytic Performance

**Figure 7:**
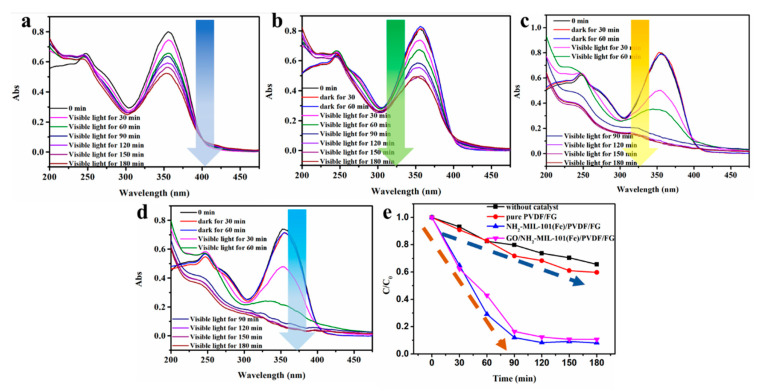
Degradation curve of TC (**a**) without catalyst, and (**b**) by pure PVDF/FG membrane, (**c**) NH_2_-MIL-101(Fe)/PVDF/FG membrane, and (**d**) GO/NH_2_-MIL-101(Fe)/PVDF/FG membrane; (**e**) comparison of degradation rate by different materials.

The photocatalytic performance on TC degradation in water was evaluated by UV-vis absorption spectra. The original TC solution exhibited a strong absorption peak at 350 nm. Firstly, the degradation of TC without catalyst was studied. As shown in Figure 7a, the results indicated that 40% degradation was achieved within 180 min with the absence of photocatalysts upon visible-light irradiation. For comparison, the degradation efficiency of the PVDF/FG membrane (Figure 7b), NH_2_-MIL-101(Fe)/PVDF/FG membrane (Figure 7c), and GO/NH_2_-MIL-101(Fe)/PVDF/FG membrane (Figure 7d) toward TC in water. In the beginning, in order to ensure adsorption equilibrium of TC onto the surface of samples, the solution of TC containing the three kinds of membranes were placed in the dark for 1 h. It can be seen from the experimental results that the sequence of degradation efficiency of the three membranes is GO/NH_2_-MIL-101(Fe)/PVDF/FG > NH_2_-MIL-101(Fe)/PVDF/FG > PVDF/FG (Figure 7e). The PVDF/FG almost has no degradation efficiency. The existence of GO has a slight contribution to the degradation efficiency of the final membrane due to its fast carrier transfer efficiency. Under the action of GO/NH_2_-MIL-101(Fe)/PVDF/FG membrane, the degradation efficiency of TC can achieve around 91% after 90 min, which is slightly higher than that of pure NH_2_-MIL-101(Fe) powdery catalysts proving that the activity of the NH_2_-MIL-101(Fe) is not affected by the morphology at all (Figure S5).

**Figure 8:**
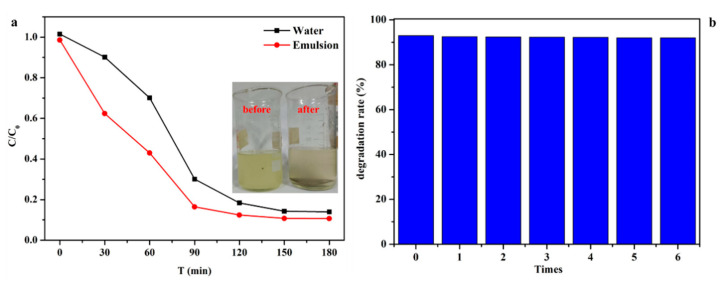
(**a**) Effect of GO/NH_2_-MIL-101(Fe)/PVDF/FG membrane on the photocatalytic degradation rate of TC in pure water and emulsion; (**b**) cycle experiment on the photocatalytic degradation rate of TC by GO/NH_2_-MIL-101(Fe)/PVDF/FG membrane.

In many cases, TC usually exists in oil-water effluents because of the continuous increase of domestic and industrial oily effluents discharge, as well as frequent leakage. Consequently, in order to remove TC from complex wastewater, we should be committed to the design of more advanced degradable materials, which are not only of scientific significance but also of practical significance. Figure 8a shows that the photocatalytic degradation rate of TC in the emulsion is higher than that in pure water, which is attributed to the membrane’s hydrophobic property. Therefore, the composite membrane has excellent photocatalytic degradation effect on tetracycline in sewage in practical application. As all know, the existence of emulsified oil droplets will reduce the reaction rate to a certain extent and may even pollute the catalytic sites [45,46]. Therefore, we also studied the reusability of the membrane for the degradation of TC. When the photocatalytic experiment is repeated five times, the degradation rate of the membrane is always stable above 90% with multiple cycles (Figure 8b). Moreover, this membrane kept its entire shape after multiple cycles without cracking, demonstrating its good mechanical strength.

#### 3.2.3. Photocatalytic Mechanism of Composite Membrane

**Figure 9:**
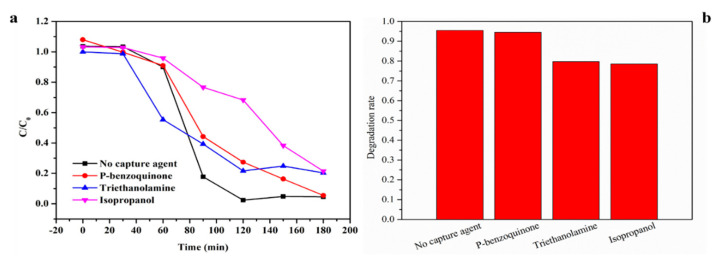
(**a**) Curves and (**b**) bar graphs of the influence of various free radical traps on the reduction rate of tetracycline.

In order to explore the role of free radicals in the photocatalytic degradation of tetracycline using composite membranes, several groups of free radical capture experiments were carried out under the same visible light irradiation conditions. In previous studies [47,48], p-benzoquinone (BQ) is a trapping agent for superoxide radicals (•O_2_^−^), triethanolamine is a trapping agent for holes (h^+^_VB_), and isopropanol is a hydroxyl radical (•OH) capture agent. Figure 9 shows the concentration ratio curve and degradation rate curve of tetracycline degradation by adding different free radical capture agents under the same conditions as the above experiment. The results indicate the effect of adding triethanolamine and isopropanol. The degradation of TC has a more obvious impact. The addition of triethanolamine reduces the degradation rate from 96.09% to 79.69%, and the addition of isopropanol reduced the degradation rate of tetracycline from 96.09% to 78.51%. Para-benzoquinone does not have much effect on it, and the degradation rate after adding p-benzoquinone is 94.51%. It can be proved that the main components of photocatalytic degradation are holes (h^+^_VB_) and hydroxyl radicals (•OH) [49].

In recent years, graphene-based materials have been widely concerned by scientific circles because of their excellent physical and chemical stability and large specific surface area. From the previous research, it can be obtained that graphene oxide (GO) as a kind of graphene-based material can combine with other semiconductors to form a direct Z-scheme photocatalyst [50], which enormously reduces the electron-hole recombination, thus improving the efficiency of photogenerated electron-hole separation. At the same time, this greatly increases the oxidation-reduction potential of the catalyst [51], thereby improving the photocatalytic performance. In GO/NH_2_-MIL-101(Fe), NH_2_-MIL-101(Fe) nanoparticles possess semiconductor properties, and its valence band oxidation is very strong. Therefore, NH_2_-MIL-101(Fe) and GO form a direct Z-scheme photocatalyst to degrade tetracycline.

**Figure 10:**
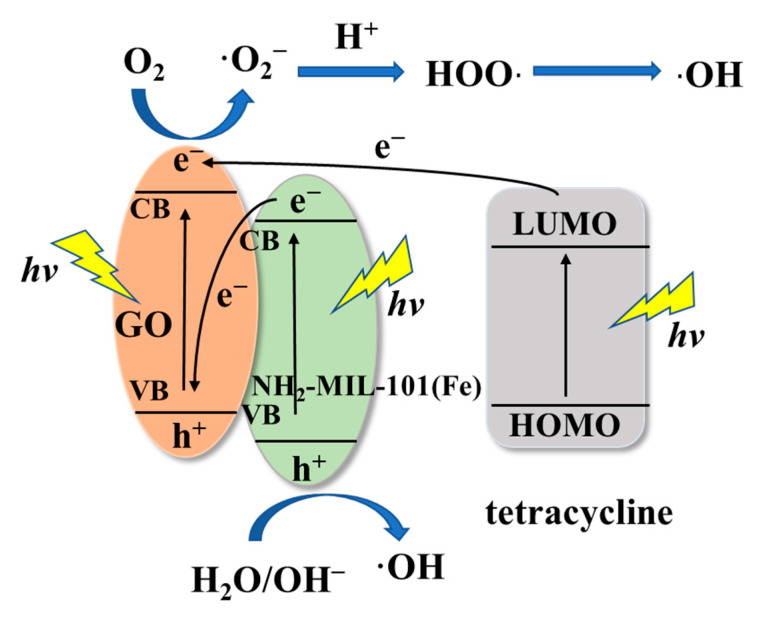
Mechanism of GO/NH_2_-MIL-101(Fe) photocatalytic degradation of tetracycline.

According to a large number of previous studies [52,53] and our experimental results, we reasonably speculate the mechanism. The reaction mechanism is shown in Figure 10. When tetracycline is excited by visible light, electrons transition from HOMO to LUMO, and photogenerated electrons can transition from LUMO to the conduction band of graphene oxide. At the same time, the ground state electrons of GO and NH_2_-MIL-101(Fe) are excited, and electrons transition from the valence band to conduction band, producing photogenerated electrons and holes. The valence band of GO can accept photogenerated electrons from the conduction band of NH_2_-MIL-101(Fe), thereby increasing the overall redox potential.

We have obtained from experiments that in the process of photocatalytic degradation of tetracycline, •OH and h^+^_VB_ are the main active species, and •O_2_^−^ is not the main active participant in the photocatalytic reaction. Therefore, we explain its photocatalytic mechanism as follows. On the one hand, on the reduction potential of GO, the O_2_ dissolved in the solution captures the photogenerated electrons on the conduction band of graphene oxide to generate superoxide radicals •O_2_^−^, and then through and Protons combine to generate HOO• radicals [54], and then capture electrons to generate peroxides, but peroxides are unstable and eventually generate OH [55]. The reaction process is as follows:

HOO• + H^+^ + e^−^ → H_2_O_2_

H_2_O_2_ + e^−^ → •OH + OH^−^

Tetracycline + •OH → Degradation

On the other hand, the holes in the valence band of NH_2_-MIL-101(Fe) have strong oxidizability, which can oxidize tetracycline molecules and directly destroy tetracycline structure. At the same time, it reacts with H_2_O/OH^−^ to generate •OH, which can also oxidize tetracycline into small inorganic molecules [56], such as H_2_O and CO_2_, etc.

#### 3.2.4. Oil-Water Separation Performance

**Figure 11:**
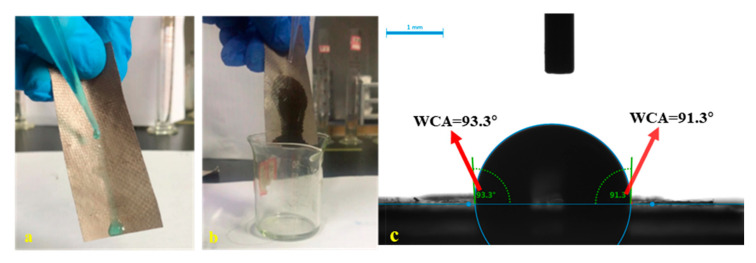
Macroscopic view of hydrophilic (**a**) and hydrophobic (**b**) composite membrane; (**c**) contact angle of the final membrane.

The hydrophobicity is a significant factor influencing the photocatalytic properties of the composite membrane. Hydrophobicity of composite membrane is the premise of oil-water separation. From the reported literature, the water contact angle of pure PVDF is 113° [57,58,59]. However, when the hydrophilic GO/NH_2_-MIL-101(Fe) was coated on it, the water contact angle of the composite membrane decreased to 93.3°. In addition, Figure 11a,b also show that the composite membrane has good hydrophobicity and lipophilicity in macroscopic performance (Video S3 and Video S4).

**Figure 12:**
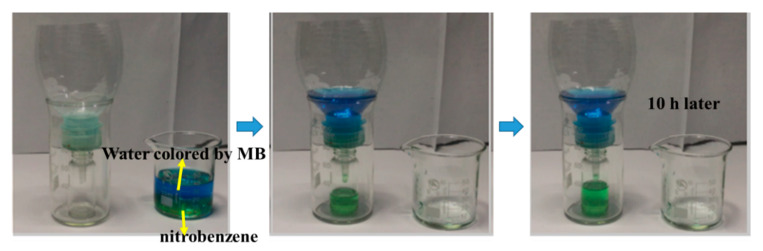
The separation test of the water/oil emulsion via a gravity-driven process.

**Figure 13:**
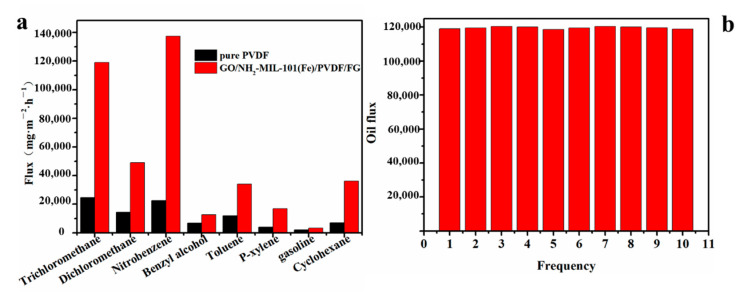
(**a**) Separation performances of the pure PVDF and GO/NH_2_-MIL-101(Fe)/PVDF/FG membranes in different oil/water mixtures; (**b**) repeatability tests of GO/NH_2_-MIL-101(Fe)/PVDF/FG membrane in memtrichloromethane/water mixture.

As shown in Figure 12, in order to assess the emulsion separation ability of the membrane, a simple device was made, which showed the separation of water/oil emulsion through the process of gravity drive. The separation performances of different membranes in various organic phase/water emulsions were evaluated by the separation capacity and flux [60,61,62], which is shown in Figure 13a. The separation efficiency was calculated as follows:

η = (m_1_/m_0_) × 100%

where m_1_ and m_0_ are the oil mass of filtered and initial emulsion, respectively. The flux was calculated by the equation:

F = V/(A × T)

where V (L) represents the filtered oil volume, A (m^2^) is the area of the membrane, and T (h) is the separating time [63].

Comparing with the pristine PVDF membrane, the flux of GO/NH_2_-MIL-101(Fe)/PVDF/FG membrane increases obviously whether in heavy-oil/water mixture or light-oil/water mixture. (Video S5 and Video S6). Additionally, the reusability of GO/NH_2_-MIL-101(Fe)/PVDF/FG membrane was also assessed by separating trichloromethane/water mixture, as shown in Figure 13b. The flux of GO/NH_2_-MIL-101(Fe)/PVDF/FG membrane almost does not change even after 10 cycles, indicating its superior recyclability.

In addition, because the composite membrane containing multiple components is difficult to replicate accurately, we re-characterized the photocatalytic performance and oil flux performance of the composite membrane with five kinds of membranes. The error and error bars are shown in Figure S6. The results show that the composite membrane has good repeatability. In conclusion, a simple method to obtain a composite membrane is introduced, that is, a coating solution containing various functional components is prepared and coated on glass fiber. The method is simple, and the effect is obvious. However, there is still room for improvement, that is, although the coating method can keep the appearance of the membrane flat, it is difficult to ensure the uniformity of the membrane thickness.

## 4. Conclusions

In order to improve the mechanical strength, a practical membrane was constructed with glass fiber as the skeleton, aiming at solving the problem of tetracycline catalyst recycling and enhancing the anti-fouling property of the PVDF membrane during oil-water separation. In accordance with the research results, it is precisely because of the existence of NH_2_-MIL-101(Fe) in the prepared membrane and the hydrophobicity of PVDF that it has good catalytic performance in the emulsion. The degradation rate for TC can achieve around 93% within 90 min in emulsion and is still higher than 90% after several cycles, while the degradation rate in pure water was 82%. No cracks were found after several cycles. The experimental mechanism studies indicate that •OH and h^+^_VB_ play significant roles in the degradation process. Moreover, after 10 cycles, the composite membrane still showed high separation efficiency and stable flux. The oil flux of the composite membrane was always stable at 12,000 mg·m^−^^2^·h^−^^1^, indicating that its high oil-resistance property and excellent recyclability. Moreover, after 10 cycles, there was no obvious change in the appearance of the material. Thus, the composite membrane has excellent cycle performance and can meet the requirements of long-term operation. Therefore, this material has a potential application prospect in the treatment of antibiotics and oil pollution in oily sewage.

## Figures and Tables

**Table 1:** The tension of the membrane.

Times	The Pure PVDF Membrane (Pa)	The PVDF/FG Membrane (Pa)	GO/NH_2_-MIL-101(Fe)/PVDF /FG Membrane (Pa)
1	18,667.47	49,159.87	49,563.14
2	24,024.38	49,022.61	49,574.25
3	23,664.90	49,029.15	49,287.32
4	27,760.35	49,290.59	49,659.87
5	28,381.93	49,227.84	49,297.13
average	24,499.81	49,146.01	49,476.34

## Data Availability

All data in this study will be available from the corresponding author upon reasonable request.

## Supplementary Materials

The following are available online at https://www.mdpi.com/article/10.3390/nano11123201/s1, Figure S1: Stability of emulsion, Figure S2: (a) The original glass fiber; (b) PVDF membrane without glass fiber; (c) PVDF membrane (with glass fiber); (d) The final membrane, Figure S3: SEM images of cross sections of (a) PVDF membrane, (b) glass fiber membrane, (c) GO/PVDF/FG membrane and (d) GO/NH2-MIL-101(Fe)/PVDF membrane at three magnifications (20, 5, 2 μm), Figure S4: The XPS spectrum of GO/NH2-MIL-101(Fe), Figure S5: Photocatalytic degradation curves of TC by NH2-MIL-101(Fe), Figure S6: (a) Photocatalytic degradation of tetracycline and (b) oil flux performance of different membranes, Video S1: Experimental study on breaking strength of PVDF/FG membrane, Video S2: Experimental study on breaking strength of GO/NH_2_-MIL-101(Fe)/PVDF/FG membrane, Video S3: Hydrophobicity test of the final membrane, Video S4: Lipophilicity test of the final membrane, Video S5: Oil flux test of the final membrane in light-oil/water mixture, Video S6: Oil flux test of the final membrane in heavy-oil/water mixture.
