# Supplementary material for: A Robust PVDF-Assisted Composite Membrane for Tetracycline Degradation in Emulsion and Oil-Water Separation
Source: Nanomaterials (Basel). 2021 Nov 26;11(12):3201. doi: 10.3390/nano11123201 (PMC8703638; doi:10.3390/nano11123201)
Supplement: Supplementary file 1 [file nanomaterials-11-03201-s001.zip › Supplementary File/Supporting information.pdf]

A robust PVDF-assisted composite membrane for tetracycline degradation in emulsion and oil-water separation

Huijun Li [a], Xin Xu [a], Jiwei Wang [a], Xuefeng Han [b,*], Zhouqing Xu [a,*]

[a]College of Chemistry and Chemical Engineering, Henan Polytechnic University, Jiaozuo, Henan 454000, China, zhqxu@hpu.edu.cn.

[b]School of Safety Science and Engineering, Henan Polytechnic University, Jiaozuo 454000, PR China, xfhan668@163.com.

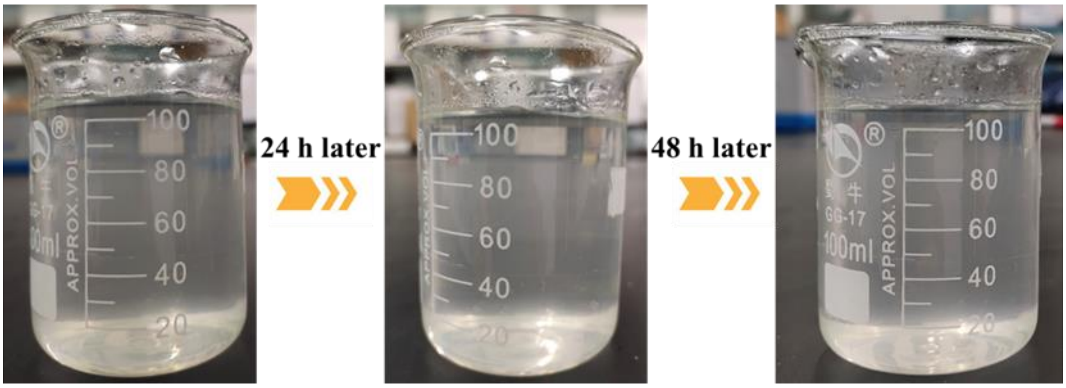

Figure S1 Stability of emulsion

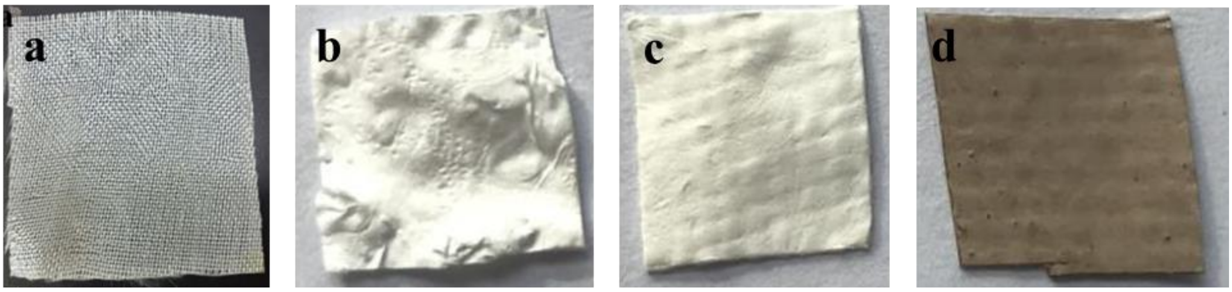

Figure S2 (a) The original glass fiber; (b) PVDF membrane without glass fiber; (c) PVDF membrane (with glass fiber); (d) The final membrane.

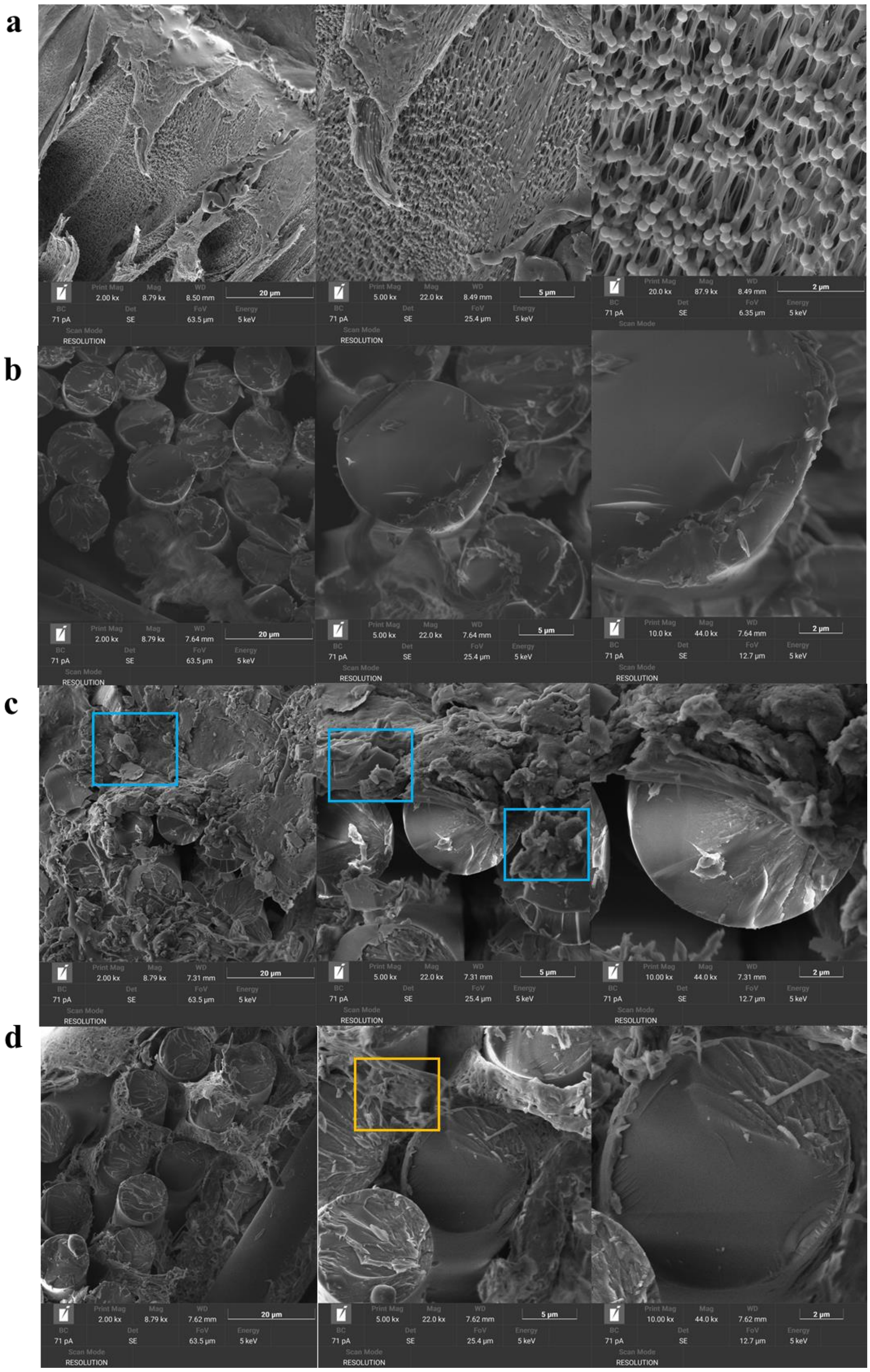

Figure S3 SEM images of cross sections of (a) PVDF membrane, (b) glass fiber membrane, (c) GO/PVDF/FG membrane and (d) GO/NH_2-MIL-101(Fe)/PVDF membrane at three magnifications (20, 5, 2 μm).

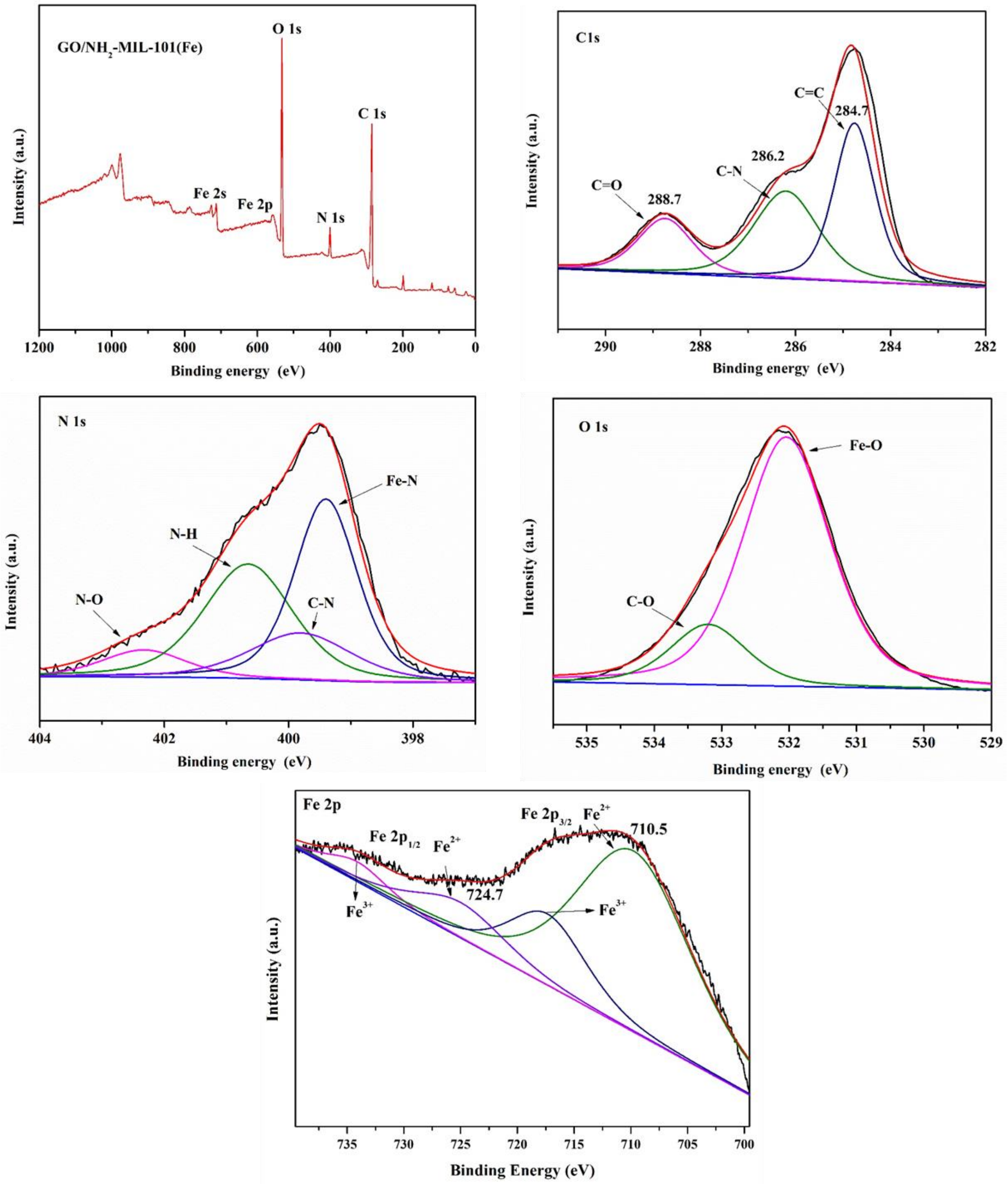

Figure S4 The XPS spectrum of GO/NH_2-MIL-101(Fe).

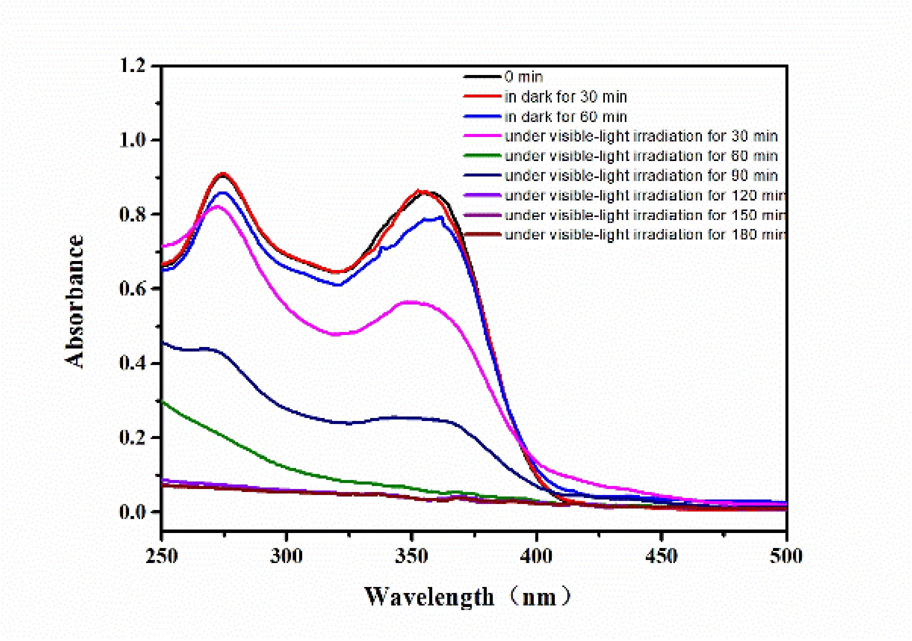

Figure S5 Photocatalytic degradation curves of TC by NH_2-MIL-101(Fe).

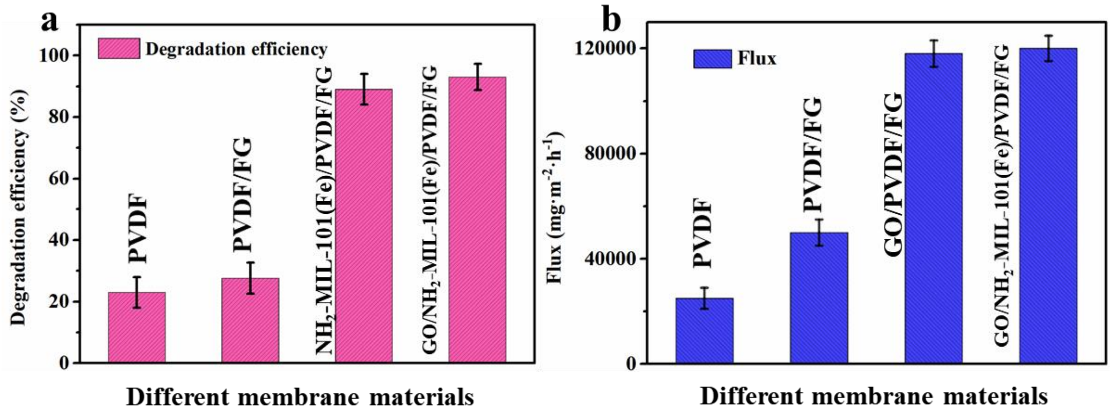

Figure S6 (a) Photocatalytic degradation of tetracycline and (b) oil flux performance of different membranes.
